# 

**Published:** 1895-12

**Authors:** 


					DECEMBER, 1895.
Vol. XIII. No. 50.
\ THE
BRISTOL
MEDICO-CHIRURGICAL
C0'V6'%;
JOURNAL,
1902
n.Vf.u:
B (Sluarterlg Journal of tbe /ifteDfcal Sciences for tbe "WHest
of England an& Soutb Males
PUBLISHED UNDER THE AUSPICES OF
THE BRISTOL MEDICO-CHIRURGICAL SOCIETT.
"Scire est ncscire, nisi 16 me
Scire alius sciret."
Editor: R. SHINGLETON SMITH, M.D., B.Sc.
WITH WHOM ARE ASSOCIATED
J. MICHELL CLARKE, M.A., M.D.
F. R. CROSS, M.B. and W. H. HARSANT.
Assistant-Editor: L. M. GRIFFITHS.
Editorial Secretary: B. M. H. ROGERS, B.A., M.D., B.Ch.
BRISTOL: J. W. ARROWSMITH ;
London : J. & A. Churchill, 11 New Burlington Street.
Price One Shilling and Sixpence.

				

